# Effect of lateral femoral cutaneous nerve-block on pain after total hip arthroplasty: a randomised, blinded, placebo-controlled trial

**DOI:** 10.1186/s12871-016-0183-4

**Published:** 2016-03-23

**Authors:** Kasper H. Thybo, Harald Schmidt, Daniel Hägi-Pedersen

**Affiliations:** 1 Department of Anaesthesiology, Næstved Hospital, Ringstedgade 61, 4700 Næstved, Denmark; 2 Department of Orthopaedic Surgery, Næstved Hospital, Ringstedgade 61, 4700 Næstved, Denmark

**Keywords:** Peripheral nerve block, Postoperative analgesia, Total hip arthroplasty, Lateral femoral cutaneous nerve block

## Abstract

**Background:**

Total hip arthroplasty (THA) is a common procedure associated with moderate postoperative pain. No nerve block without loss of motor function has been documented for THA. We hypothesised that an ultrasound-guided lateral femoral cutaneous nerve (LFCN) block added to a multimodal postoperative pain regimen would reduce postoperative pain after THA.

**Methods:**

One hundred patients who had a THA by the posterior approach were evaluated in this randomised, placebo-controlled, blinded, parallel-group trial comparing an ultrasound-guided LFCN-block with either 8 ml of ropivacaine, 7.5 mg/ml, (Group Ropivacaine) or 8 ml of saline (Group Placebo) given postoperatively. Surgery was performed under spinal anaesthesia. The primary outcome was pain (measured on a Visual Analogue Scale (VAS)) 4 h post-blockade during 30° flexion of the hip. Secondary outcomes were pain at rest, pain during movement, oxycodone consumption (0–24 h), time to mobilisation, ability to mobilise, and length of stay. Patients, assessors and all staff involved with patient care were blinded to the intervention.

**Results:**

There was no difference in primary outcome between Group Ropivacaine and Group Placebo (VAS 27 mm vs. 31 mm, *p* = 0.41; difference −5 mm (95 % CI: −15 mm - +5 mm). No differences in any of the secondary outcomes were observed. No adverse events, or harms, were observed during the trial.

**Conclusion:**

Pain scores, opioid use, time to mobilisation, and length of stay were low in both Group Ropivacaine and Group Placebo. We found no added analgesic effect of a LFCN-block when combined with paracetamol and ibuprofen after THA by the posterior approach.

**Trial registration:**

EudraCT: 2013-004501-12 (December 16th 2013)

## Background

There is no gold standard for pain management after total hip arthroplasty (THA) [1, 2]. Early mobilisation is the main priority [3] and different combinations of non-opioid drugs, peripheral nerve blocks, epidural analgesia and local infiltration analgesia (LIA) are used to reduce opioid requirements and opioid-related adverse effects. THA is a very common procedure and most patients are elderly and thus have a lower tolerance of opioid related side-effects.

Wound pain may play a role in pain after THA. It has been demonstrated that the size of the incision is directly related to postoperative pain and minimal incision THA is shown to reduce postoperative pain [4, 5]. Local wound infiltration has been used as part of an analgesic regimen for THA, but its place in postoperative pain management is debated [6–8]. A recent systemic review concludes that LIA have limited additional analgesic efficacy in THA when combined with a multimodal analgesic regimen consisting of paracetamol, celecoxib and gabapentin [6].

Peripheral nerve blocks used for postoperative pain management after THA include femoral nerve block, fascia iliaca block and lumbar plexus block [1]. All have analgesic effects but these peripheral nerve blocks are associated with motor blockade and may lead to falling [9]. The priority of early mobilisation warrants a pure sensory block.

The lateral femoral cutaneous nerve (LFCN) is a sensory branch from the lumbar plexus and has a highly variable course and supplies parts of the lateral and anterior upper thigh [10–15]. The LFCN-block is a pure sensory block and seeks to remove wound pain after THA. Ultrasonic guided LFCN-block has never been investigated as a part of multimodal analgesic regimen for THA.

The aim of this placebo controlled trial was to investigate the effects of LFCN-block on pain, opioid requirements and mobilisation in patients receiving a basic analgesic regimen consisting of paracetamol and ibuprofen after THA. We hypothesised that LFCN-block would reduce pain during movement 4 h after LFCN-block (primary outcome) without delaying mobilisation.

## Methods

This prospective, randomised, placebo-controlled trial was approved by the Danish Medicine Agency, (EudraCT registration number: 2013-004501-12, December 16th 2013), the local Regional Ethics Committee, Region Zealand, Allén 15, 4180 Sorø, (SJ-367, Chairperson: Knud Rasmussen, on December 16th 2013) and the Danish Data Protection Agency. The Copenhagen University Good Clinical Practice Unit monitored the trial. The trial was conducted at Næstved Hospital, Næstved, Denmark and registered at clinicaltrials.gov (NCT02289937) on November 12th 2014.

All patients provided written informed consent before participating. Eligible participants were patients scheduled for primary THA under spinal anaesthesia. Exclusion criteria were general anaesthesia, allergy against local anaesthetics, revision arthroplasty, bilateral arthroplasty, fertile women and patients with daily use of opioids.

Randomisation was based on a computer-generated randomisation list, in a ratio of 1:1. The randomisation list and sealed, opaque envelopes were made by a secretary with no further involvement in this trial. Upon inclusion, subjects received treatment assigned according to the randomisation list, in consecutive numbered, opaque, sealed envelopes.

The study medication was prepared by a nurse according to the randomisation list. This was verified by a second nurse. These nurses were not further involved in treating the patient, or in the trial. The study medication was prepared in a syringe and labelled with the patient’s id-number and the number according to the randomisation list. Ropivacaine and saline are visually indistinguishable.

All investigators, patients, outcome assessors, and clinical personnel were blinded to the intervention.

All subjects received a primary hip arthroplasty. This was either cemented, uncemented or hybrid using the posterior approach. No LIA was used. The subjects were anaesthetised with spinal anaesthesia induced with 2–2.5 ml isobaric 0.5 % bupivacaine. Sedation with propofol or remifentanil was administered at the discretion of the anaesthesiologist. In the case of failure of the spinal anaesthesia and conversion to general anaesthesia the participant would be removed from the trial and replaced.

The LFCN-blocks were performed in the post anaesthesia care unit when the subjects were able to move their toes but before the spinal anaesthesia had worn off (defined as T0). The subjects received an active or placebo LFCN-block according to randomisation. The LFCN-blocks were performed by anaesthesiologists specially trained and certified to the procedure.

**Fig. 1:**
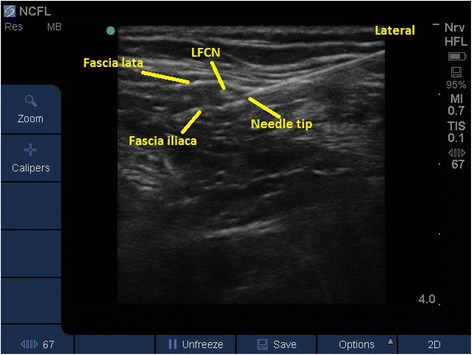
Ultrasound image of the lateral femoral cutaneous nerve. LFCN, Lateral Femoral Cutaneous Nerve

A high-frequency linear ultrasound transducer (15–6 MHz) was used to scan the area (Sonosite S-nerve). The study medicine was administrated as a bolus of 8 ml 7.5 mg/ml ropivacaine or 8 ml isotonic saline. We identified the LFCN between the fascia lata and the fascia iliaca (Fig. 1) by scanning lateral-to-medial from the anterior superior iliac spine along the inguinal ligament. Before needle insertion the femoral nerve, vein and artery were located to avoid complications. The needle (B-Braun Ultraplex, 22G × 80 mm) was introduced in-plane from lateral-to-medial. Correct needle placement was confirmed with bolus’ of 1–2 ml saline at the discretion of the anaesthesiologist. The correct spread of the bolus around the LFCN was observed.

Postoperative pain treatment consisted of 1 g of paracetamol administrated orally at 6 h intervals and 600 mg of ibuprofen administrated orally at 8 h intervals initiated immediately after the surgery. Opioids were administrated by the nurses if the patients scored more than 30 mm on a Visual Analogue Scale (VAS), or when requested by the patient. The opioid used was oxycodone, either orally or intravenous.

The primary outcome was difference in pain between the groups during 30 ° flexion of the hip at four hours after T0 (T4). Secondary outcomes were pain at rest and during 30 ° flexion of the hip at T0, T1, T2, T4, T8, T12 and T24 (corresponding 0, 1, 2, 4, 8, 12, and 24 h after T0), cumulative oxycodone consumption (0–24 h postoperatively), time to first administration of oxycodone, length of hospital stay, time to mobilisation and ability to mobilise.

We assessed pain with the VAS-score (0–100 mm; 0 mm, no pain; 100 mm, worst pain imaginable) at rest and during 30 ° active flexion of the hip. The mobilisation ability was assessed using the Cumulated Ambulation Score (CAS; 0–6, 0, no mobilisation; 6 fully mobilised) [16, 17]. Opioid administrations were collected from the electronic patient chart.

### Data handling and statistics

A difference of 20 mm in VAS-scores at T4 during 30 ° flexion of the hip between the two groups was considered clinical relevant. From the literature we found a standard deviation (SD) of 30 mm [18]. With a type I error (alfa) of 5 % and a power (1 – beta) of 80 %, 2 × 36 patients were needed. To account for the uncertainty of the true SD and to gain more power we included 2 × 50 evaluable patients.

Data were analysed using IBM SPSS Statistics version 21 for Windows (SPSS, Chicago, Illinois, USA). We used Shapiro-Wilks test to test for normality of our data. Data are presented as mean and SD, or median and first and third quartile as appropriate. For comparison of our non-parametric data we used Mann-Whitney U-test and the Hodges-Lehman estimator. A two-sided *p* < 0.05 was considered statistical significant. Bonferroni correction was used for multiple comparisons.

After completion of the trial the data were typed into a spreadsheet by two investigators and compared for typos. A randomisation list assigning subjects to either group “a” or “b” was provided without revealing the identity of the groups. The statistical analysis had been performed and conclusions were drawn before it was revealed which group was ropivacaine and which was placebo.

## Results

**Fig. 2:**
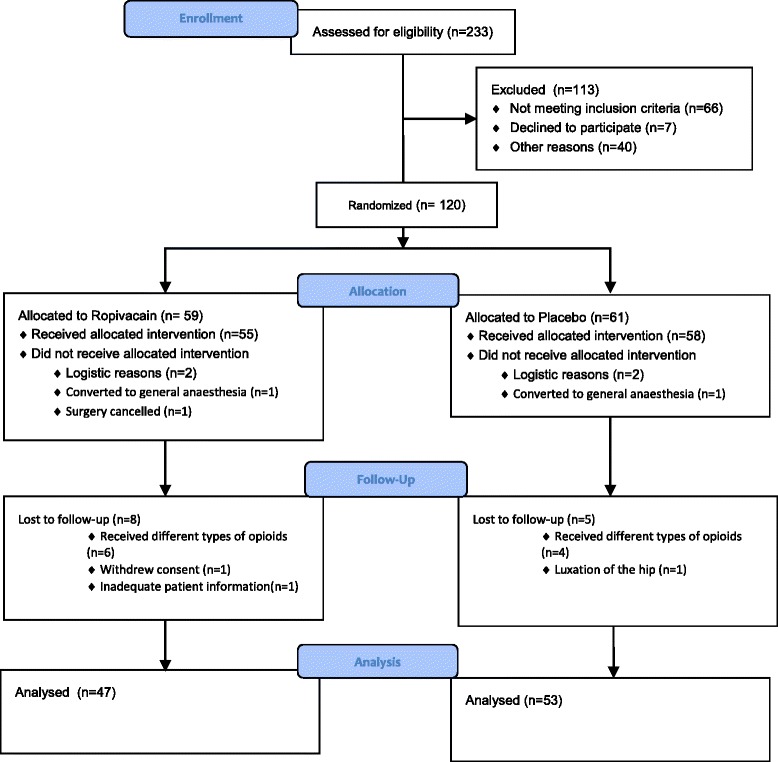
Flow-chart

One hundred twenty subjects were enrolled in the study from 3rd of March 2014 to 1st of October 2014 in order to reach the pre-specified number of 100 patients because 20 did not complete the study (Fig. 2). Thus, one hundred subjects were evaluated (47 in Group Ropivacaine and 53 in Group Placebo). The subjects’ characteristics did not differ between the two groups (Table 1).

**Table 1:** Basic demographics

	Group ropivacaine	Group placebo
No patients	47	53
Gender, M	23	25
Age, years	69 (11)	69 (8)
Height, cm	170 (10)	171 (10)
Weight, kg	79 (18)	78 (14)
ASA		
1	16	24
2	27	28
3	4	1
Duration of surgery, min	62 (16)	61 (17)
Duration from end of surgery to block, min	63 (28)	65 (28)

Age, height, weight, duration of surgery, and duration from end of surgery to block are mean (SD)

Twenty subjects were excluded from the trial for various reasons. These were distributed equally between the ropivacaine and placebo group. Reasons for exclusion were administration of opioids other than prescribed in the study protocol, inadequate spinal anaesthesia with conversion to general anaesthesia, or daily use of opioids prior to surgery that the patient neglected to inform the investigators about until after the surgery.

The VAS-scores at T4 during 30 ° flexion of the hip were 31 and 26.5 mm in the Group Placebo and the Group Ropivacaine, respectively, and the difference was −5 mm (95 % CI: −15 - +5 mm), *p* = 0.41.

**Fig. 3:**
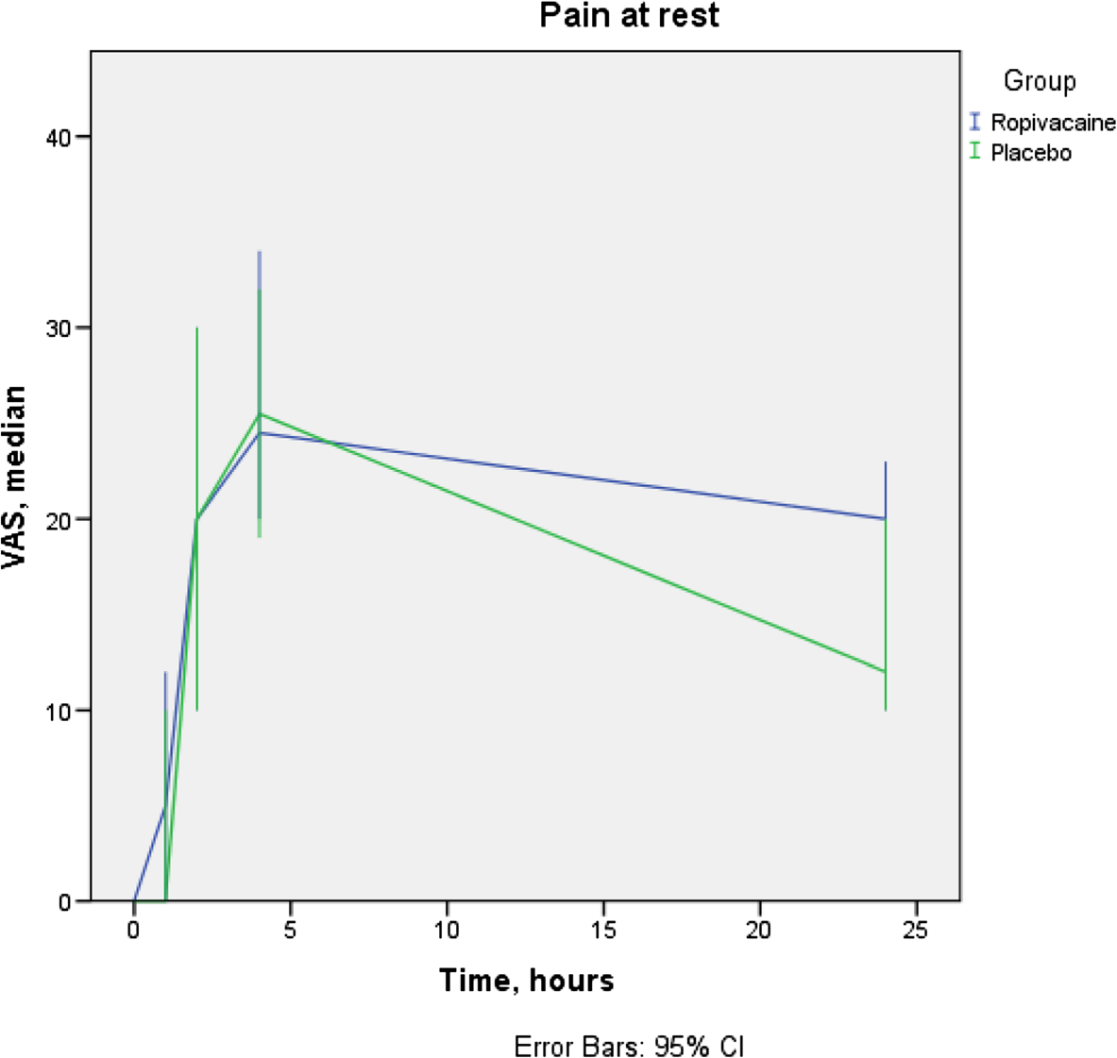
Pain at rest. For T0, T1, T2, T4, and T24 there are 0, 9, 5, 4, and 16 % missing data respectively, equally distributed between groups. No differences were statistical significant. VAS, Visual Analogue Scale

**Fig. 4:**
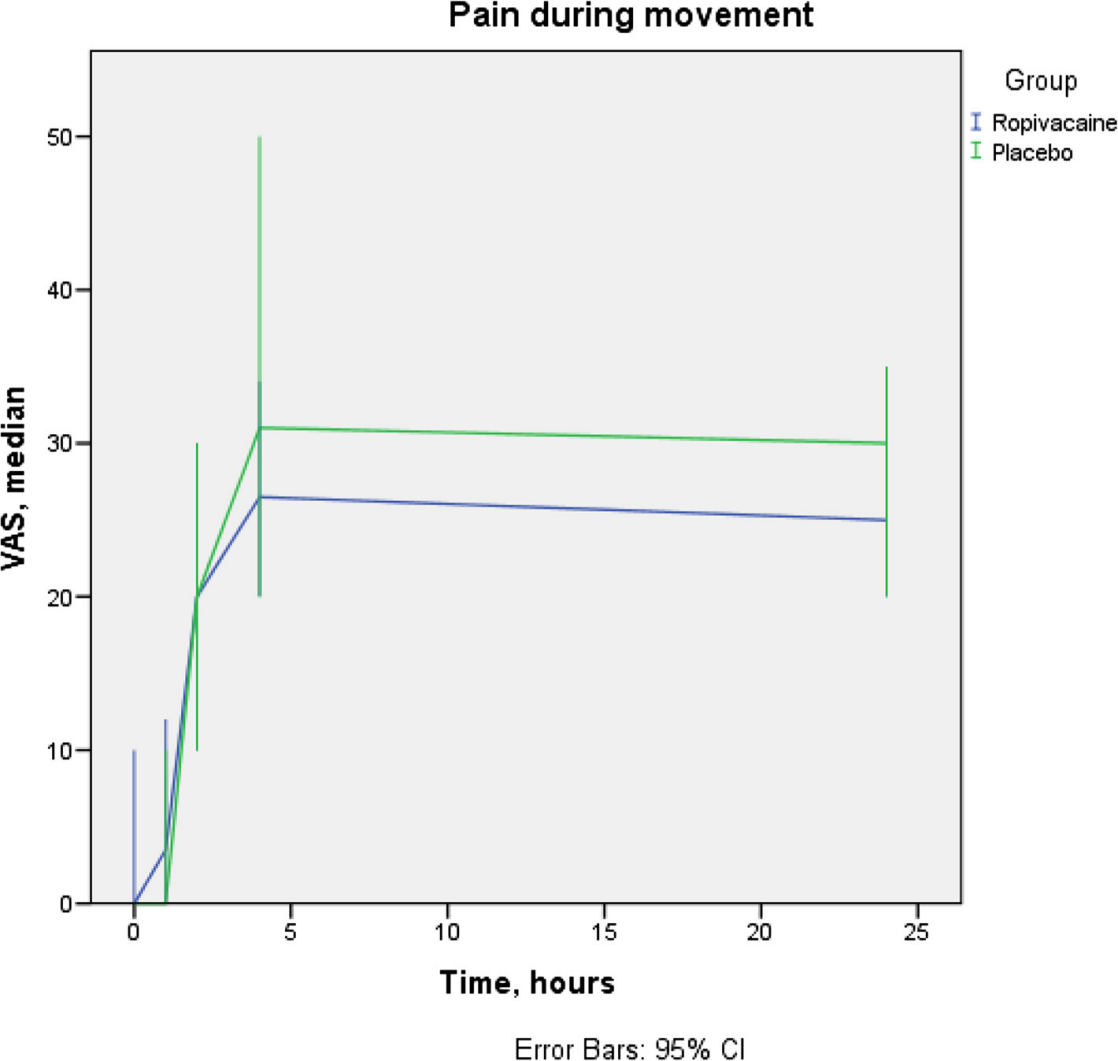
Pain during movement. For T0, T1, T2, T4, and T24 there are 8, 18, 18, 14, and 28 % missing data respectively, equally distributed between groups. No differences were statistical significant. VAS, Visual Analogue Scale

Due to unforeseen difficulties obtaining data for all subject we had only few VAS-scores for T8 and T12. Data for VAS-scores are seen in Figs. 3 and 4.

Oral oxycodone was converted to intravenous oxycodone (20 mg/7,5 mg) (http://pro.medicin.dk/Laegemiddelgrupper/Grupper/227010#a300). The median intravenous-oxycodone consumption was 7 mg (2–13) and 6 mg (2–9), *p* = 0.12 in the ropivacaine group and the placebo group, respectively. There was no difference in the time to first opioid-administration between the two groups (Table 2).

**Table 2:** Outcomes

	Group Ropivacaine	Group Placebo median	
Oxycodone consumed the first 24 h, mg	7(2–13)	6 (2–9)	*P* = 0.12
Time to first oxycodone demand, min	253 (144–379)	237 (155–380)	*P* = 1
Cumulated ambulation score the first day	5 (3–6)	5 (4–6)	*P* =0.26
Time from surgery to first ambulation, min	368 (330–519)	420 (364–470)	*P* = 0.69
Length of hospital stay, hours	49 (10)	50 (20)	*P* = 0.53

Values are median and upper and lower quartile or mean and standard deviation. P-values are Mann Whitney U-test

There were missing values for 40 subjects with respect to the mobility assessment (equally distributed between groups). Of the remaining 60 subjects there were no differences in mobilisation ability (CAS = 5 in both groups, *p* = 0.26) or time to first mobilisation (368 vs. 420 min, *p* = 0.69).

There were no adverse effects related to the study medication.

## Discussion

This randomised, blinded, placebo-controlled trial comparing the analgesic effect of lateral femoral cutaneous nerve block on pain after total hip arthroplasty by the posterior approach showed no differences between the two groups regarding pain scores, neither during movement, nor at rest, during the first 24 h postoperatively. In addition we observed no differences in opioid requirements. To our knowledge, this is the first prospective trial to evaluate ultrasound-guided LFCN-block for THA.

The median pain scores, both during movement and at rest, were low in both groups, which was unexpected. In our search of the literature we found a median VAS-score during walking of 50 mm the first postoperative day [19, 20]. Moderate pain corresponds to VAS-score = 30 mm and is considered to provide adequate sensitivity in acute pain trials [21–23]. The median pain score during flexion of the hip at 4 h postoperatively in our control group is barely “moderate pain”, and perhaps our trial did not have sufficient sensitivity to detect the effect of any analgesic intervention.

All subjects in this trial received a basic analgesic regimen with paracetamol, ibuprofen and oxycodone at request. This could explain the low pain scores in both groups and a possible conclusion of this trial could be that this basic analgesic regimen is sufficient for the majority of patients after THA. We cannot rule out any benefits in those patients with more severe pain, and thus further studies are needed to explore this aspect.

Despite the fact that our sample size calculation was based on a power of 80 % we do not believe we have missed any relevant effects of the LFCN-block in this setting because we decided to include additional patients to account for the uncertainty of the SD and to give the trial more power. We believe that 100 subjects are sufficient for this type of trial.

Our study was challenged by a rather large number of missing data for pain scores at T8 and T12 (40–60 %). The main reason for this was unforeseen staff shortage on the ward. Furthermore, many patients were asleep and we decided not to assign a specific VAS-score to a sleeping patient. We have chosen not to show the pain scores for these time points and not to calculate the Area Under the Curve (AUC) for the first 24 h because of the lack of data. This is an obvious drawback of this study, but we believe that it does not alter the conclusion of our study due to the fact that we observed no differences at T0, T1, T2, T4, and T24.

Another explanation for the lack of significant differences in pain is block failure. No sensory testing was performed in this trial. Sensory testing is routine practice at our institution, but we decided not to perform this in order to avoid unblinding the trial. Block failure is a rather unlikely explanation because the LFCN-block is fairly easy to perform, with a high success rate and was executed by few anaesthesiologists who were trained and certified for the procedure.

A number of anatomical variations of LFCN has been described [10–13], and we cannot be sure that the area supplied by the LFCN covered the incision in our trial. A recent study by Davies et al. [14] suggests that LFCN-block may not cover the expected line of incision for THA by posterior approach in a large proportion of cases. Davies et al. concludes that the incision may be too posterior and extent too superior to be covered by the LFCN.

## Conclusion

In conclusion, we demonstrated no additional analgesic effects of a lateral femoral cutaneous nerve-block when combined with a basic analgesic regimen with paracetamol, ibuprofen and oxycodone after THA by posterior approach. Further studies are needed to investigate the effect of the LFCN-block among patients with higher pain scores.

## Abbreviations

LFCN: lateral femoral cutaneous nerve
LIA: local infiltration analgesia
SD: standard deviation
THA: total hip arthroplasty
